# Perioperative cognitive changes in older adults undergoing major non‐cardiac surgery

**DOI:** 10.1002/alz70856_107638

**Published:** 2026-01-09

**Authors:** Anaelle Charles, Mili Jocelyn Jimenez Gallardo, Andrea Castillo Suarez, Elizabeth Sugg, Peng Li, Kun Hu, Lei Gao

**Affiliations:** ^1^ Perioperative Sleep and Brain Health Lab, Massachusetts General Hospital, Boston, MA, USA; ^2^ Harvard Medical School, Boston, MA, USA; ^3^ Medical Biodynamics Center, Massachusetts General Hospital, Boston, MA, USA; ^4^ Massachusetts General Hospital, Boston, MA, USA

## Abstract

**Background:**

Approximately one‐third of adults over the age of 65 undergoing surgery experience severe cognitive impairments, including acute confusion, attention deficits, and global cognitive dysfunction. Older adults with greater cognitive impairments are at increased risk for prolonged hospitalization, higher rates of readmission, institutionalization, and long‐term cognitive decline, including dementia and Alzheimer's disease. However, the trajectory of postoperative cognitive changes in older adults remains poorly understood. Cognitive assessments such as the *Montreal Cognitive Assessment* (MOCA) are commonly used in clinical settings but are often interpreted as a single total score, overlooking domain‐specific cognitive changes. To address this research gap, this study examines domain‐specific cognitive changes in older surgical patients before and after surgery.

**Method:**

Seventeen older surgical patients (≥70 years old) undergoing knee, hip, or spine surgery were recruited. Cognitive function was assessed pre‐ and post‐surgery using the Montreal Cognitive Assessment (MOCA). Assessments were captured 1‐week before surgery and 1‐day post‐surgery. A linear mixed‐effects model was used to evaluate cognitive function pre‐ and post‐surgery across the following cognitive domains, abstraction, attention, language, orientation, and recall.

**Result:**

The analysis revealed an interaction effect between event group (pre‐ vs. post‐surgery) and the MOCA cognitive domains (*F*(4,126) = 6.97, *p* < 0.01), indicating that post‐operative cognitive functions possibly decline at different rates across cognitive domains. *Attention, orientation*, and *recall* demonstrated the strongest effects, where the *recall* domain showed significant decline following surgery compared to baseline (*β* = ‐0.87 (*standardized*), *SE* = 0.156, *p* < 0.01), suggesting a domain‐specific vulnerability in memory function.

**Conclusion:**

Our findings indicate that postoperative cognitive decline is domain‐specific, with memory function exhibiting the greatest vulnerability and possibly a slower recovery trajectory. These results suggest that assessing cognitive domains individually, rather than relying solely on total MOCA scores, may advance early detection of patients at risk for prolonged cognitive impairment. Follow‐up studies are warranted to determine whether domain‐specific cognitive assessments can serve as predictors of long‐term cognitive decline, including postoperative delirium and dementia, ultimately guiding early interventions to improve patient outcomes.

This research was supported by AACSF‐23‐1148490

